# Chloroplast Lipids Metabolism and Function. A Redox Perspective

**DOI:** 10.3389/fpls.2021.712022

**Published:** 2021-08-05

**Authors:** M. Luisa Hernández, Francisco Javier Cejudo

**Affiliations:** Instituto de Bioquímica Vegetal y Fotosíntesis, Universidad de Sevilla-Consejo Superior de Investigaciones Científicas, Sevilla, Spain

**Keywords:** chloroplast, fatty acid, ferredoxin, lipid, membrane, redox regulation

## Abstract

Plant productivity is determined by the conversion of solar energy into biomass through oxygenic photosynthesis, a process performed by protein-cofactor complexes including photosystems (PS) II and I, and ATP synthase. These complexes are embedded in chloroplast thylakoid membrane lipids, which thus function as structural support of the photosynthetic machinery and provide the lipid matrix to avoid free ion diffusion. The lipid and fatty acid composition of thylakoid membranes are unique in chloroplasts and cyanobacteria, which implies that these molecules are specifically required in oxygenic photosynthesis. Indeed, there is extensive evidence supporting a relevant function of glycerolipids in chloroplast biogenesis and photosynthetic efficiency in response to environmental stimuli, such as light and temperature. The rapid acclimation of higher plants to environmental changes is largely based on thiol-based redox regulation and the disulphide reductase activity thioredoxins (Trxs), which are reduced by ferredoxin (Fdx) *via* an Fdx-dependent Trx reductase. In addition, chloroplasts harbour an NADPH-dependent Trx reductase C, which allows the use of NADPH to maintain the redox homeostasis of the organelle. Here, we summarise the current knowledge of chloroplast lipid metabolism and the function of these molecules as structural basis of the complex membrane network of the organelle. Furthermore, we discuss evidence supporting the relevant role of lipids in chloroplast biogenesis and photosynthetic performance in response to environmental cues in which the redox state of the organelle plays a relevant role.

## Introduction

In oxygenic photosynthesis, ferredoxin (Fdx), at the stromal side of photosystem I (PSI), functions as a mobile carrier distributing reducing equivalents from the photosynthetic electron transport chain to produce NADPH, *via* Fdx-NADP-reductase (FNR), or to thioredoxins (Trx), *via* Fdx-Trx-reductase (FTR; Cejudo et al., 2019). Trxs participate in the light-dependent reductive activation of biosynthetic enzymes, including Calvin-Benson cycle enzymes (Michelet et al., 2013), among other processes. In addition, chloroplasts harbour a NADPH-dependent Trx reductase termed NTRC (Serrato et al., 2004). NTRC is an efficient reductant of 2-Cys peroxiredoxin (Prx; Moon et al., 2006; Pérez-Ruiz et al., 2006; Pérez-Ruiz and Cejudo, 2009), which suggested an antioxidant function for the enzyme. However, the Arabidopsis mutant devoid of NTRC shows impaired light-dependent reduction of enzymes of the Calvin-Benson cycle (Thormählen et al., 2015; Ojeda et al., 2017), indicating a function in redox regulation for the enzyme. More recently, it was proposed that NTRC and 2-Cys Prxs form a redox relay that modulates the reducing capacity of Trxs allowing the light-dependent activation of their downstream targets (Pérez-Ruiz et al., 2017). Thus, a tight functional relationship exists between chloroplast redox regulation and thiol-dependent antioxidant systems (Cejudo et al., 2021).

Chloroplast thylakoid membranes have a characteristic lipid composition and content of unsaturated fatty acids. Lipids have a well-established structural function serving as matrix for the photosynthetic complexes and allowing the compartmentalisation of the organelle, but also affect chloroplast biogenesis and photosynthetic performance. Environmental cues, such as light and temperature changes, affect chloroplast lipids biosynthesis and fatty acids desaturation, a process influenced by the redox state of the organelle (Geigenberger and Fernie, 2014; Yu et al., 2020). In this review, we summarise the lipid biosynthetic pathways in the chloroplast and the current knowledge of the role of lipids in chloroplast biogenesis and performance. The function of lipids in thylakoid membrane biogenesis and the organisation of membrane-associated processes during chloroplast differentiation is discussed. Moreover, the role of the unsaturated fatty acid content of thylakoid membranes on the photosynthetic activity in response to environmental cues is also updated. Finally, we discuss the redox regulatory mechanisms that control lipids biosynthesis and fatty acids desaturation, thus coordinating the redox state with chloroplast performance and plant growth.

## Chloroplast Fatty Acid and Glycerolipids Biosynthetic Pathways

**Figure 1:**
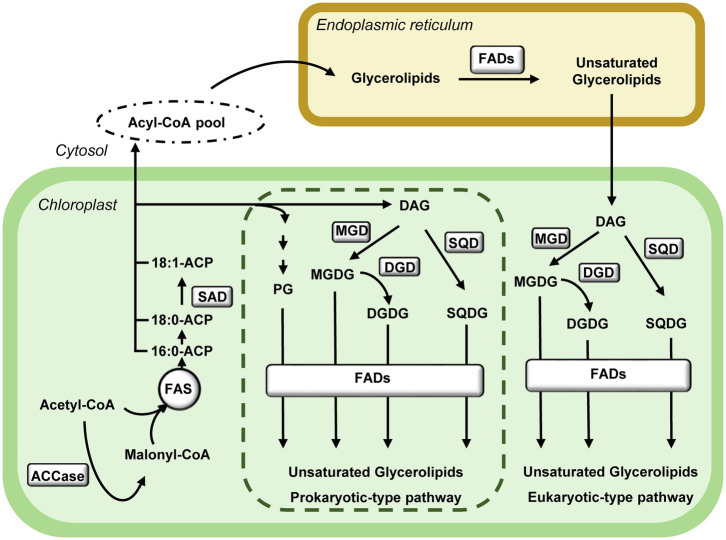
Pathways of glycerolipids biosynthesis in Arabidopsis. *De novo* synthesised fatty acids in chloroplasts are incorporated into lipids *via* two pathways. The prokaryotic-type pathway takes place exclusively in the chloroplast (dotted box), the eukaryotic-type pathway takes place in the endoplasmic reticulum and the chloroplast using the glycerol backbone assembled in the endoplasmic reticulum. The synthesis of polyunsaturated fatty acids is catalysed by membrane-bound fatty acid desaturases (FADs) in both cell compartments. ACCase, Acetyl-CoA carboxylase; DAG, diacylglycerol; DGDG, digalactosyldiacylglycerol; DGD, DGDG synthase; FADs, membrane-bound fatty acid desaturases; FAS, Fatty acid synthase complex; MGDG, monogalactosyldiacylglycerol; MGD, MGDG synthase; PG, phosphatidylglycerol; SAD, stearoyl-ACP desaturase; SQDG, sulfoquinovosyldiacylglycerol; and SQD, SQDG synthase.

**Figure 2:**
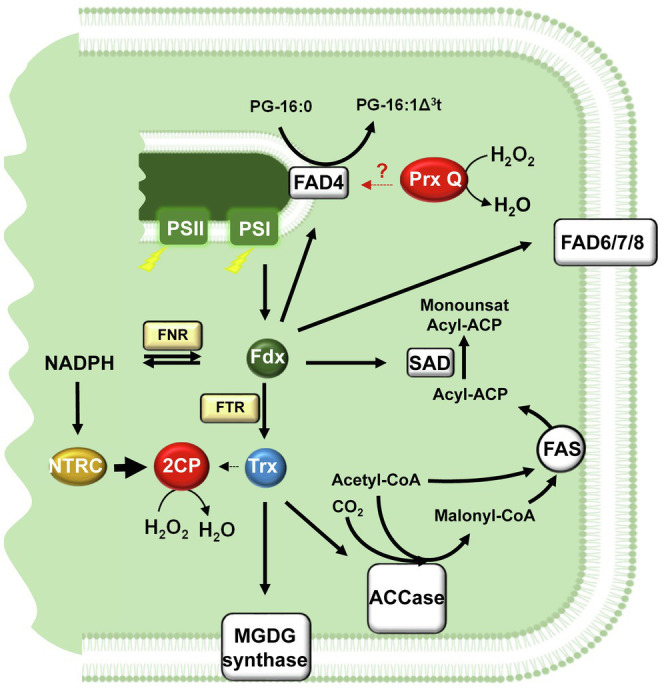
Redox control of chloroplast lipid metabolism. Fdx, the final acceptor of the photosynthetic electron transport chain, acts as a hub distributing reducing equivalents for the formation of NADPH *via* FNR, to enzymes involved in fatty acid desaturation, SAD and FADs and to Trxs *via* FTR. Initial reactions of lipid biosynthesis catalysed by ACCase and MGDG synthase undergo Trx-dependent redox regulation. The reductive capacity of chloroplast Trxs is balanced by the NTRC-2-Cys Prxs redox relay, which uses NADPH as source of reducing power and hydrogen peroxide as final sink of electrons. An additional chloroplast Prx, Prx Q, affects FAD4 activity at thylakoid membrane, though the molecular basis of this effect is not yet known. ACCase, Acetyl-CoA carboxylase; 2CP, 2-Cys Prx; FAD4, phosphatidylglycerol desaturase (palmitate specific); FAD6, chloroplast omega-6 desaturase; FAD7/8, chloroplast omega-3 desaturases; FAS, Fatty acid synthase complex; FNR, Fdx-NADP reductase; FTR, Fdx-dependent Trx reductase; Fdx, ferredoxin; MGDG, monogalactosyldiacylglycerol; NTRC, NADPH-dependent Trx reductase C; Prx, peroxiredoxin; SAD, stearoyl-ACP desaturase; and Trx, thioredoxin.

*De novo* plastid fatty acid biosynthesis is initiated by the ATP consuming conversion of acetyl-CoA and CO_2_ to malonyl-CoA catalysed by acetyl-CoA carboxylase (ACCase). Malonyl-CoA is the substrate of the fatty acid synthase (FAS) complex, which performs the consecutive condensation of acetyl-CoA units to generate palmitoyl-acyl carrier protein (16:0-ACP) and stearoyl-ACP (18:0-ACP) as main products (Ohlrogge and Browse, 1995; Figure 1). Fatty acid desaturation is initiated by the soluble stearoyl-ACP desaturase (SAD) that forms oleoyl-ACP (18:1-ACP), as main product of plastid fatty acid biosynthesis. Fatty acids are then incorporated into the two plant glycerolipids biosynthesis pathways, prokaryotic- and eukaryotic-type (Figure 1). The prokaryotic-type pathway is exclusive of plastids and involves the synthesis of phosphatidylglycerol (PG), and the glycolipids monogalactosyldiacylglycerol (MGDG), digalactosyldiacylglycerol (DGDG) and sulfoquinovosyldiacylglycerol (SQDG). Alternatively, fatty acids can be exported to the cytosol and incorporated into the eukaryotic-type pathway. Part of the glycerolipids assembled in the endoplasmic reticulum return to the plastid to serve as substrates for glycolipids synthesis (Li-Beisson et al., 2013; Figure 1). In both pathways, fatty acids can be further desaturated by the membrane-bound fatty acid desaturases (FAD). Chloroplast FADs use glycerolipids as substrates and reduced Fdx, produced by the photosynthetic electron transport chain in the light or from NADPH in the dark, as electron donor (Shanklin and Cahoon, 1998; Figure 2).

## Glycerolipids Affect Chloroplast Biogenesis and Photosynthetic Function

The fact that the lipid composition of thylakoid membranes is unique and highly conserved suggests a specific requirement of these lipids for the structure and function of photosynthetic complexes (Petroutsos et al., 2014). The most abundant lipids in thylakoid membranes are galactolipids MGDGs and DGDGs, which account for about 50 and 25% of total thylakoid lipids, respectively (Douce and Joyard, 1996), the sulfolipids SQDGs and the phospholipids PGs. MGDGs are non-bilayer forming lipids with conical shape that participate in thylakoid curvature, whereas DGDGs have cylindrical shape, are bilayer forming and were proposed to stabilise the membrane lipid bilayer (Jouhet, 2013; Garab et al., 2017). Thus, for photosynthetic membranes stability, the MGDG/DGDG ratio is crucial (Demé et al., 2014). SQDGs and PGs are bilayer forming lipids that contain negatively charged head groups. The amount of total anionic lipids in the thylakoid membranes is strictly maintained by compensation between SQDGs and PGs contents. X-ray crystallographic studies identified integral glycolipids specifically bound to the core proteins of photosynthetic complexes that participate in protein-protein and protein-cofactors interactions (Kern et al., 2009). These findings support the notion that, besides providing the structural matrix for photosynthetic complexes, lipids participate in the formation of the photosynthetic machinery during chloroplast biogenesis.

MGDGs, the most abundant glycolipids in thylakoid membranes, are essential for chloroplast structure and photosynthetic performance (Jarvis et al., 2000; Kobayashi et al., 2007). MGDGs synthesis is catalysed by MGDG synthases (MGD); then, DGDGs are synthesised by the galactosylation of MGDGs catalysed by DGDG synthases (DGD; Figure 1; Boudière et al., 2014). Arabidopsis mutants with residual amounts of DGDGs show impaired photosynthetic performance (Kelly et al., 2003) and PSI alteration (Ivanov et al., 2006), while mutants deficient in MGDGs show impaired formation and maintenance of PSI and PSII (Kobayashi et al., 2013; Fujii et al., 2014), indicating their relevant role in chloroplast structure and function. This notion was further confirmed by *in vitro* studies showing the specific requirement of MGDGs for the ordered oligomerization of light harvesting complex II (LHCII; Schaller et al., 2011), the dimerisation of PSII (Kansy et al., 2014) and the coupling between LHCII and PSII core complexes (Zhou et al., 2009).

An additional effect of thylakoid lipids is exerted on gene expression as shown by the impaired expression of plastid and nuclear photosynthesis-related genes in the Arabidopsis *mgd1-2* mutant (Kobayashi et al., 2013). Thus, a transcriptional link between the synthesis of chlorophyll, photosynthetic proteins, and galactolipids seems to be essential for the organisation of the thylakoid membrane networks (Kobayashi et al., 2014). Consistent with these data, the expression of *MGD1* gene was downregulated in Arabidopsis PG deficient mutants, which failed to develop functional chloroplasts (Kobayashi et al., 2015). Thus, MGD1 activity might link galactolipid synthesis with chloroplast transcriptional regulation during cotyledon greening (Fujii et al., 2014). Once MGDGs are properly synthesised during chloroplast biogenesis, the development of chloroplast progresses even if *MGD1* expression is inhibited afterwards, indicating that MGDGs synthesis is essential at early stages of the process (Fujii et al., 2014).

The etioplast-to-chloroplast differentiation upon illumination involves the transformation of prolamellar bodies (PLBs) and prothylakoids of etioplasts in fully organised thylakoid membranes (Solymosi and Schoefs, 2010; Kowalewska et al., 2016, 2019). Etioplasts lack chlorophyll but accumulate the chlorophyll precursor protochlorophyllide (Pchlide; Lebedev et al., 1985; Schoefs, 2001) and have similar lipid composition than thylakoid membranes (Selstam and Sandelius, 1984). Galactolipids have different functions in chlorophyll biosynthesis and organisation of light-harvesting complexes during the etioplast-to-chloroplast transition (Fujii et al., 2019b). MGDGs are involved in Mg-Proto IX metabolism for Pchlide biosynthesis and are essential for the formation of Pchlide-LPOR-NADPH ternary complexes in PLBs, which are responsible for the Pchlide to Childe conversion upon illumination (Fujii et al., 2017, 2019a). On the other hand, DGDGs are required for the conversion of Mg-Proto IX methyl ester to Pchlide (Fujii et al., 2018). Furthermore, MGDGs, but not DGDGs, enhance the oligomerization of the Pchlide-LPOR complex, whereas DGDGs play a specific role in the dissociation of Childe-LPOR complex and the formation of PLBs structure (Gabruk et al., 2017; Fujii et al., 2018). Galactolipids have also different contributions to the development of the thylakoid membranes during chloroplast differentiation. The Arabidopsis *mgd1* knockout mutant shows totally blocked thylakoid biogenesis indicating that MGDGs are necessary for grana formation and stacking (Kobayashi et al., 2007). On the other hand, although DGDGs also contribute to grana stacking, the Arabidopsis *dgd1-2* mutant, severely deficient in DGDGs, exhibits a slow development of bent thylakoids with highly stacked membranes (Mazur et al., 2019). Moreover, DGDGs have a critical role as galactolipid zipper during membrane stacking (Demé et al., 2014). It should be noted that the lack of *MGD1* affects DGDGs, which are synthesised from MGDGs. Therefore, both galactolipids have essential but different roles during chloroplast biogenesis.

Anionic thylakoid glycerolipids, PGs and SQDGs, also play an important role in chloroplast biogenesis acting as allosteric regulators in the formation of Pchlide-LPOR-NADPH complexes (Gabruk et al., 2017). Analysis of Arabidopsis mutants impaired in PGs biosynthesis revealed the essential role of these lipids in thylakoid membrane formation (Hagio et al., 2002; Babiychuk et al., 2003). Moreover, PGs deficiency affects electron transfer from antenna pigments to the PSII reaction centre (Kobayashi et al., 2016). These *in vivo* data agree with *in vitro* studies suggesting that depletion of PGs in thylakoid membranes impairs the function of the photosynthetic complexes (Siegenthaler et al., 1987; Kruse et al., 2000). The role of PGs cannot be substituted by glycolipids (Kobayashi et al., 2015, 2016), except under specific environmental conditions, such as phosphate starvation (Benning et al., 1993; Güler et al., 1996). Under standard growth conditions, SQDGs are not essential since the *sqd2* mutant showed normal growth and photosynthetic parameters (Yu et al., 2002). However, under phosphate limitation SQDGs substitute anionic phospholipids (PGs) to maintain the negative charge at the lipid-water interface. Under these conditions, the total content of anionic thylakoid lipids becomes limiting for chloroplast structure and function (Yu and Benning, 2003).

## Role of Lipids in Chloroplasts Thermotolerance: Photoinhibition by Temperature

Lipids of thylakoid membranes have also an unusual and characteristic fatty acid composition. Trienoic fatty acids (16:3 and 18:3) represent 60–70% of total fatty acids in thylakoids and more than 90% of the fatty acids in MGDGs, the most abundant chloroplast lipids. In addition, the atypical fatty acid Δ3-trans-hexadecenoate (16:1Δ^3^t) is present as a component of PGs, the major phospholipid in thylakoid membranes (Li-Beisson et al., 2013). Fatty acid unsaturation is determined by the activity of fatty acid desaturases (FADs), which introduce double bonds into specific acyl chain positions. Interestingly, most of the mutants affected in FADs show wild-type phenotype when grown under standard conditions (Wallis and Browse, 2002); in fact, 16:1Δ^3^t and trienoic fatty acids could be eliminated without any significant effect on photosynthesis and plant growth (Wallis and Browse, 2002). However, some mutant phenotypes that become evident under stressful conditions suggest a role of thylakoid fatty acid composition in photoinhibition.

PSII function requires the fine adjustment between D1 inactivation and replacement at the core of PSII (Baena-González and Aro, 2002). While D1 photo-damage is highly dependent on light intensity (Anderson and Chow, 2002), the recovery process depends on temperature and is highly affected by the level of unsaturation of thylakoid membrane lipids (Los et al., 2013). Temperature affects the fluidity of chloroplast membranes, which can be compensated by changes in the level of the unsaturation of their fatty acids: cold causes membrane rigidity, which can be alleviated by increasing unsaturation, whereas heat causes fluidisation, which can be amended by replacement of unsaturated fatty acids by *de novo* synthesised saturated ones (Falcone et al., 2004).

The degree of fatty acid unsaturation affects chilling sensitivity in *Nicotiana tabacum* leaves, the higher the degree of unsaturation, the lower the chilling sensitivity (Murata et al., 1992). These results were further confirmed by the finding that up to 88% of high-melting point fatty acids (16:0+18:0+16:1Δ^3^t) in PGs does not affect D1 inactivation but is important for its recovery after low-temperature photoinhibition (Moon et al., 1995). Later, Vijayan and Browse (2002) suggested that the level of high-melting point fatty acids is tightly correlated with recovery from photoinhibition at temperatures lower than 27°C. A threshold level of these PG fatty acids may be required for photoinhibition since no detectable differences between *fab1* plants, with 69% of high-melting point PG fatty acids, and wild-type plants, with only 55%, were found. Similarly, the level of trienoic fatty acids in chloroplast lipids has been related to photoinhibition recovery at low temperatures (Vijayan and Browse, 2002). Although the Arabidopsis *fad3-2fad7-2fad8* mutant, lacking 18:3 and 16:3 fatty acids, has only subtle effects on photosynthetic performance at temperatures as low as 5°C in the short-term, prolonged incubation at low temperature provoked a severe effect, revealing the essential role of trienoic fatty acids in photosynthetic capacity at low temperature (Routaboul and Browse, 2000). Consistent with these results, transgenic tomato plants overexpressing plastid omega-3 desaturase, hence having increased 18:3/18:2 ratio, showed alleviated photoinhibition under chilling conditions and higher tolerance to low temperature (Liu et al., 2008; Domínguez et al., 2010). Interestingly, analysis of the *fad3-2fad7-1fad8* triple mutant, with decreased contents of leaf trienoic fatty acids, exhibited wild-type levels of quantum yield of electron transfer (ɸ_II_) at 4°C (Routaboul and Browse, 2000). These results highlight the importance of trienoic fatty acids for chloroplast response to low temperatures, beyond maintenance of membrane fluidity. Moreover, Barkan et al. (2006) described a *fab1* suppressor line that could survive after 16 weeks at 2°C. This line was an allele of the *fad5* containing 31% of 16:0 compared to 23% in *fab1* and 17% in wild type. Thus, the suppressed line does not behave as expected since the increase in saturated fatty acids in the *fab1fad5-2* double mutant would increase sensitivity to low temperature. To explain these surprising results, it was suggested that the suppressor phenotype could be caused by a change in lipids molecular shape, which is important for several membrane functions (Wilhelm et al., 2020).

The level of thylakoid lipids unsaturation is also important for plants tolerance to high temperatures as shown by the enhanced thermotolerance of Arabidopsis mutants with reduced contents of polyunsaturated fatty acids in thylakoid membranes (Hugly et al., 1989; Routaboul et al., 2012). Similar results were obtained in *FAD7* silenced *N. benthamiana* leaves containing higher dienoic to trienoic fatty acids ratio, which show better photosynthesis performance at high temperature (Murakami et al., 2000; Hiremath et al., 2017). Routaboul et al. (2012) reported a close correlation of the thermal damage to the O_2_-evolving complex with the level of 16:3 fatty acid since mutants with lower amount of 16:3 fatty acids in thylakoid membranes lipids showed higher thermotolerance.

Altogether, these results indicate that the degree of chloroplast lipid desaturation plays a key role in plant acclimation to temperature changes; however, the relationship between thylakoid lipids, photosynthetic performance and temperature is highly complex and the molecular mechanism underlying this relationship remains to be elucidated. Evidence of chloroplast FAD regulation by temperature has been reported. The *fad7* mutant shows unaltered trienoic fatty acids at temperatures below 20°C (Browse et al., 1986), which was proposed to be due to a compensation effect exerted by the induction of the *FAD8* gene at low temperature (McConn et al., 1994; Román et al., 2015). Similarly, high temperatures affected FAD8 activity more severely than FAD7 activity (Román et al., 2015), though in this case a destabilisation of FAD8 protein was reported (Matsuda et al., 2005). Therefore, FAD8 regulation at either transcriptional or post-transcriptional levels could play a significant role in plant response to temperature. Interestingly, FAD8 showed higher specificity for PGs (Román et al., 2015), which are essential for photosynthetic complexes arrangement, development of thylakoid membranes and tolerance to chilling (Wada and Murata, 2007).

## Redox Regulation of Chloroplast Lipid Metabolism

The first committed step of fatty acid biosynthesis is catalysed by plastid ACCase, a multienzyme complex composed of biotin carboxyl carrier protein (BCPP), biotin carboxylase (BC) and carboxyltransferases (CT)-α and -β subunits (Sasaki et al., 1993). Different mechanisms participate in the control of ACCase activity by light, such as changes in stromal pH and Mg^2+^ (Sasaki et al., 1997; Ye et al., 2020a), CT-β mRNA editing (Sasaki et al., 2001), ‘envelope-docking’ (Ye et al., 2020b) and redox regulation (Sasaki and Nagaro, 2004). Moreover, ACCase is also regulated in response to long-term changes in light intensity (Yu et al., 2020), evidencing that the first step of fatty acid, which controls carbon flow into the pathway, is highly regulated (Figure 2). In pea leaves, CT activity of ACCase is redox regulated (Sun et al., 1997; Kozaki and Sasaki, 1999) resulting in light-dependent activation of fatty acid synthesis, with Trx *f* playing a more relevant role that Trx *m* in this regulatory mechanism (Sasaki et al., 1997). Site-directed mutagenesis revealed the formation of a disulphide linking Cys-267 in CT-α and Cys-442 in CT-β subunits (Kozaki et al., 2001). Interestingly, while Cys-267 is highly conserved, Cys-442 is not conserved in plants, such as spinach (Hunter and Ohlrogge, 1998) and tobacco (Ohlrogge et al., 1993), hence suggesting that an additional Cys is involved in this redox regulatory mechanism. In Arabidopsis, no redox regulation of ACCase has been described so far; however, the recent identification of the ACCase CT-β subunit as partner of NTRC suggested a putative role of the NTRC/2-Cys Prx system in the redox regulation of lipid biosynthesis in this species (González et al., 2019). Redox regulation also extends to other metabolic reactions of lipid metabolism. This is the case of galactolipid biosynthesis since MGDG synthase undergoes Trx-dependent redox regulation (Shijojima et al., 2013; Yamaryo et al., 2006; Figure 2), thus, linking the role of galactolipids in thylakoid structural reorganisations to light (Yu et al., 2020).

Recently, Horn et al. (2020) reported that FAD4 activity requires Prx Q to produce wild-type levels of 16:1Δ^3^t in Arabidopsis, although redox regulation of the enzyme was discarded. It is unlikely that Prx Q acts as electron donor for FAD4 because it does not contain any FeS cluster as Fdx, the reported electron donor for chloroplast FADs (Shanklin and Cahoon, 1998). An additional possibility is that Prx Q protects FAD4 enzyme from oxidative stress, given the role of PG 16:1Δ^3^t preventing photoinhibition under temperature stress (Murata et al., 1992; Moon et al., 1995). In fact, the protective role of antioxidant enzymes on photosynthesis under high temperature has been reported (Almeselmani et al., 2006) and, consequently, maintaining a redox homeostasis is an important common regulatory pathway for plant tolerance to temperature stress (Yuan et al., 2019).

## Concluding Remarks and Future Perspectives

The content of lipids and fatty acids in chloroplast thylakoid membranes is unique of this organelle and different from other plant cell compartments, which indicates a major role of lipids in chloroplast structure and function. In addition to the structural function of lipids to maintain the complex membranous network of chloroplasts, in this review, we have summarised evidence supporting an important role of lipids in the formation of photosynthetic complexes, which affects chloroplast biogenesis. Moreover, thylakoid membrane lipids have a deep effect on the photochemical reactions of photosynthesis. As sessile organisms, plant growth is highly affected by environmental cues, such as light and temperature, and there is strong evidence in support of the relationship of the degree of chloroplast fatty acid unsaturation with plant acclimation to temperature changes. Therefore, the complex pathways of chloroplast lipid and fatty acid biosynthesis are tightly regulated both at the transcriptional and post-transcriptional levels. Since Fdx is the electron donor for SAD and FAD activities, the redox state of the organelle directly affects the degree of fatty acid unsaturation. It was recently shown that Prx Q affects FAD4 activity; however, the mechanism of this effect is still poorly understood. Similarly, it has been shown that ACCase and MGDG synthase undergo Trx-mediated redox regulation, suggesting an important role of light in the regulation of initial steps of chloroplast lipid biosynthesis, though the molecular basis of this regulatory mechanism is not yet fully understood. The progress in the understanding of the mechanisms of control of chloroplast redox homeostasis and its relationship with thiol-dependent antioxidant systems, as well as the availability of Arabidopsis mutants affected in chloroplast redox regulation, provides an excellent opportunity to progress in elucidating the mechanisms regulating chloroplast lipid biosynthesis in response to environmental cues.

## Author Contributions

MLH and FJC contributed to the conception of the study. MLH drafted the manuscript. All authors revised, edited and approved the final submitted version.

## Conflict of Interest

The authors declare that the research was conducted in the absence of any commercial or financial relationships that could be construed as a potential conflict of interest.

## Publisher’s Note

All claims expressed in this article are solely those of the authors and do not necessarily represent those of their affiliated organizations, or those of the publisher, the editors and the reviewers. Any product that may be evaluated in this article, or claim that may be made by its manufacturer, is not guaranteed or endorsed by the publisher.
